# Supplementary material for: Cohesin Can Remain Associated with Chromosomes during DNA Replication
Source: Cell Rep. 2017 Sep 19;20(12):2749–55. doi: 10.1016/j.celrep.2017.08.092 (PMC5613076; doi:10.1016/j.celrep.2017.08.092)
Supplement: Document S1. Figures S1–S5 [file mmc1.pdf]

Cell Reports, Volume *20*

Supplemental Information

Cohesin Can Remain Associated with Chromosomes during DNA Replication

James D.P. Rhodes, Judith H.I. Haarhuis, Jonathan B. Grimm, Benjamin D. Rowland, Luke D. Lavis, and Kim A. Nasmyth

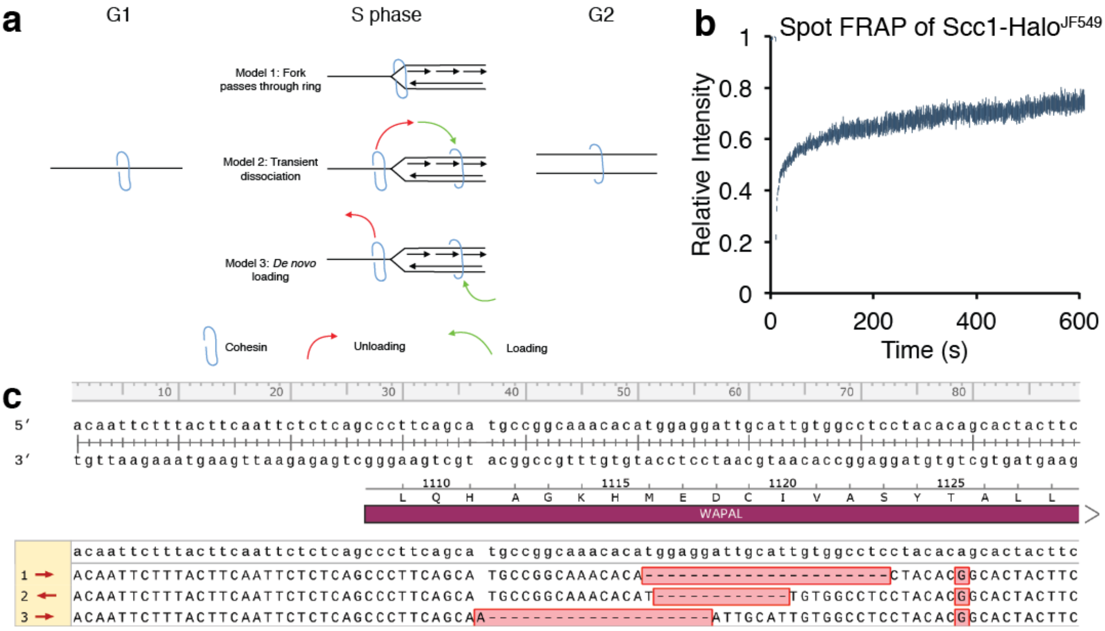

Figure S1: Related to Figure 2 **a)** Models of cohesion establishment: 1. The replication machinery passes through the cohesin ring 2. When the replication fork makes contact with cohesin, the ring transiently opens, remains associated with the replisome and reloads around sister chromatids behind the fork. 3. Cohesin is removed from chromosomes by DNA replication and cohesin is loaded de novo around sister chromatids after fork passage. **b)** Conventional spot FRAP of Scc1-Halo^{JF549} in G1 U2OS cells. n=10. Data are represented as mean ± SEM. **c)** Mutations created in *WAPL* in Scc1-Halo *WAPL*$^{1116\text{-}1119\Delta}$ U2OS cells.

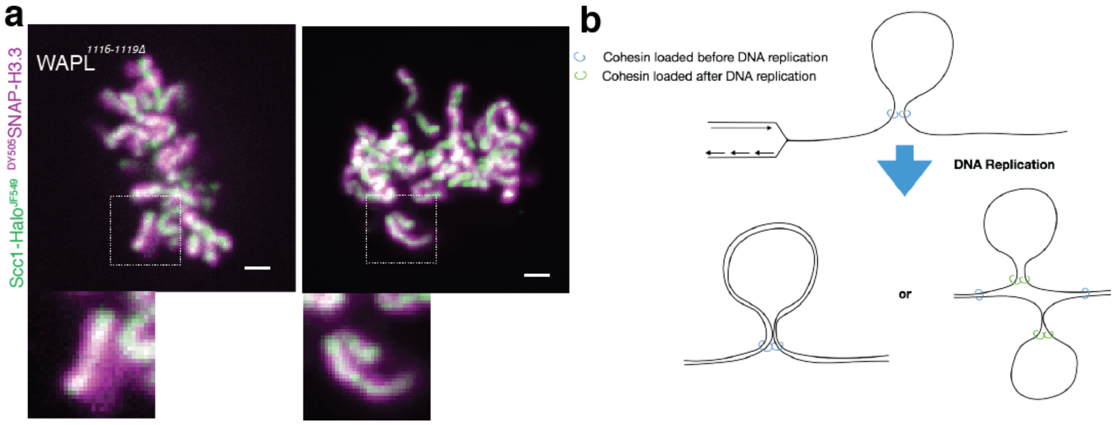

Figure S2: **Are DNA loops held by cohesive cohesin? Related to Figure 3. a)** Live cell microscopy images of Scc1-Halo^{JF549} ^{DY505}SNAP-H3.3 *WAPL*$^{1116\text{-}1119\Delta}$ U2OS cells in mitosis. Scale bar, 2 µm **b)** Models of sister chromatid cohesion in relation to DNA loops. Cohesin that is loaded before DNA replication remains associated during DNA replication and may contribute to cohesion. Cohesin in vermicelli is found between metaphase sister chromatids. This could be because cohesin can hold loops and sister chromatids together at the same time or loop extrusion may stop at established cohesin resulting in a common axis for cohesion and loops.

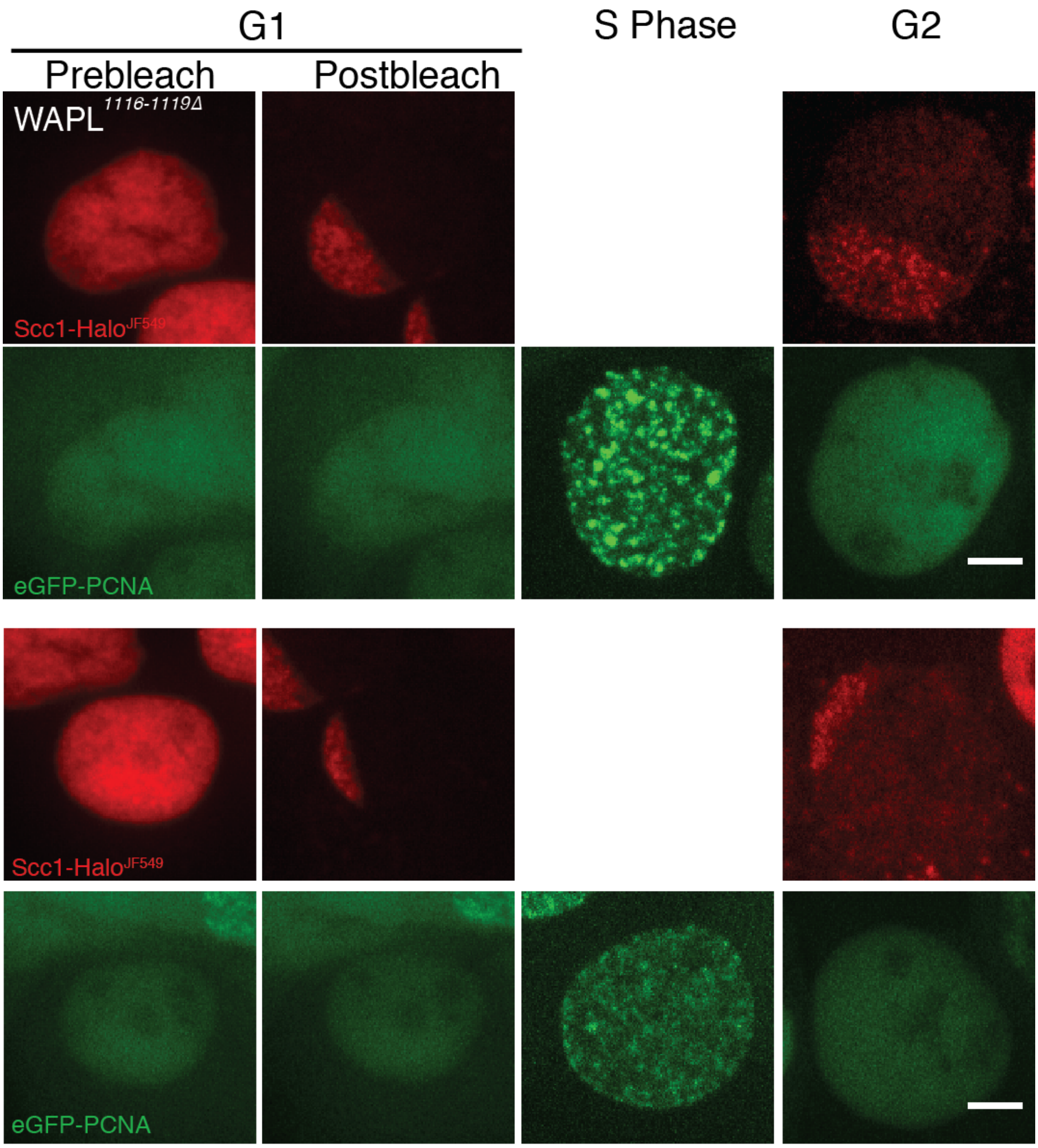

Figure S3. **Cohesin can remain associated with DNA during S-phase, Related to Figure 4.** Live cell microscopy images of Scc1-Halo^{JF549} *WAPL*$^{1116\text{-}1119\Delta}$ eGFP-PCNA cells in G1 before photobleaching, in G1 after photobleaching, in S-Phase and in G2. It was not possible to image Scc1-Halo^{JF549} between G1 and G2 because of low starting signal in G1. This fluorescence was lost easily by photobleaching from eGFP and JF549 acquisition. Contrast has been enhanced in Scc1-Halo^{JF549} channel in post bleach (after soluble fraction was bleached intentionally) and G2 (as there was a decrease in total nuclear signal). Scale bar, 5 µm.

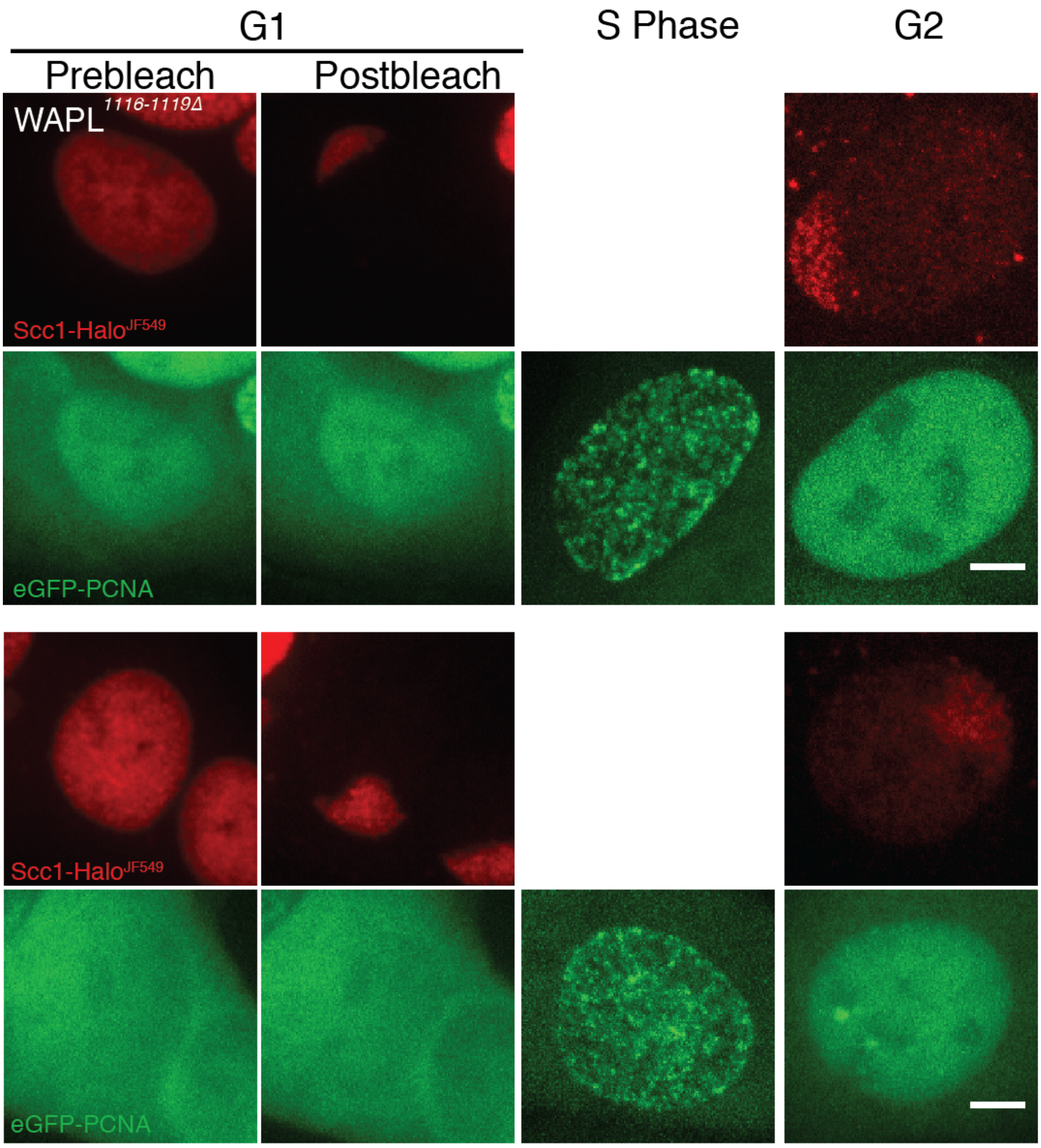

Figure S4: **Cohesin can remain associated with DNA during S-phase, Related to Figure 4.** Live cell microscopy images of Scc1-Halo^{JF549} *WAPL*$^{1116\text{-}1119\Delta}$ eGFP-PCNA cells in G1 before photobleaching, in G1 after photobleaching, in S-Phase and in G2. It was not possible to image Scc1-Halo^{JF549} between G1 and G2 because of low starting signal in G1. This fluorescence was lost easily by photobleaching from eGFP and JF549 acquisition. Contrast has been enhanced in Scc1-Halo^{JF549} channel in post bleach (after soluble fraction was bleached intentionally) and G2 (as there was a decrease in total nuclear signal). Scale bar, 5 µm.

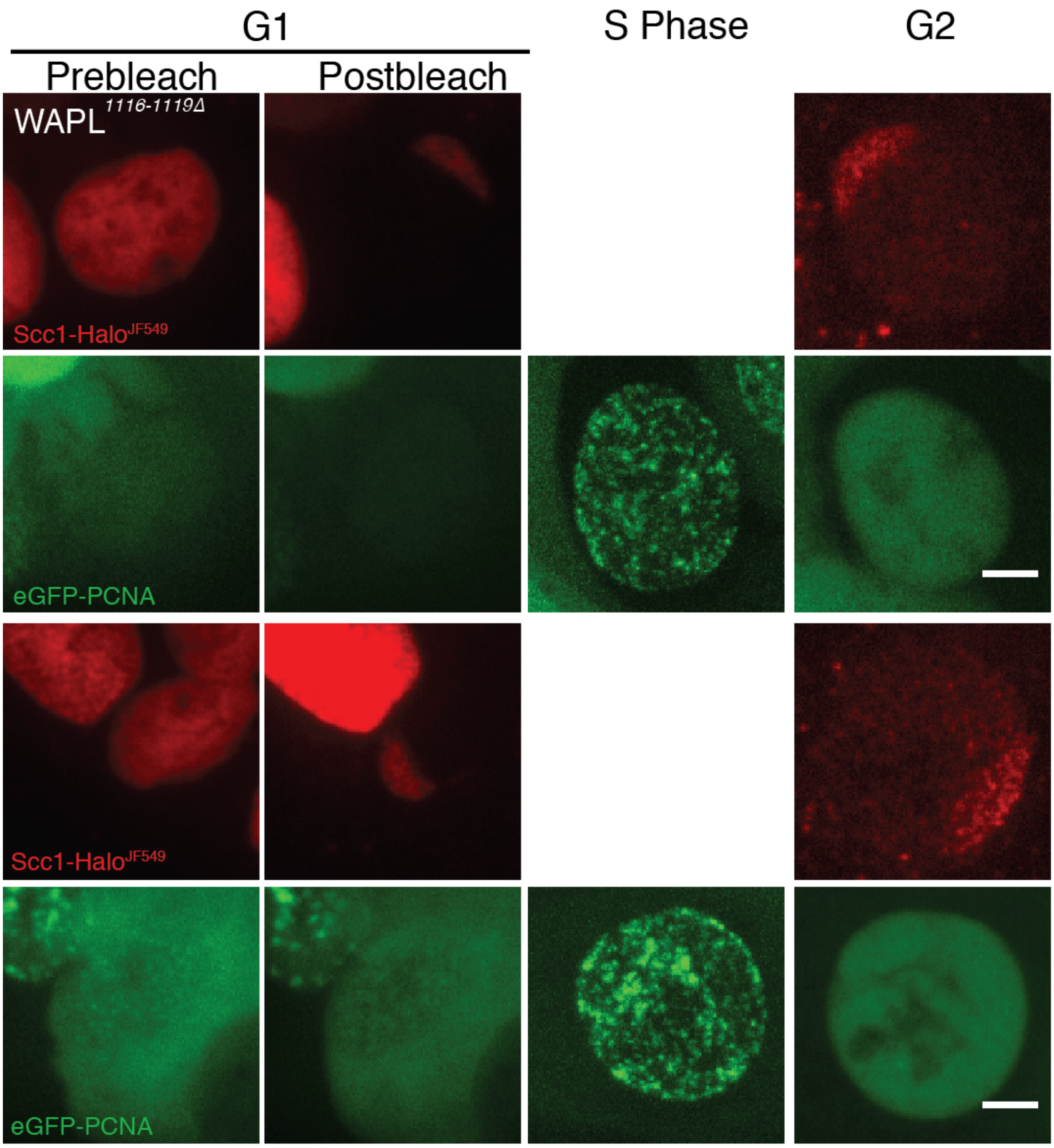

Figure S5: Cohesin can remain associated with DNA during S-phase, Related to Figure 4. Live cell microscopy images of Scc1-Halo^{JF549} *WAPL*$^{1116\text{-}1119\Delta}$ eGFP-PCNA cells in G1 before photobleaching, in G1 after photobleaching, in S-Phase and in G2. It was not possible to image Scc1-Halo^{JF549} between G1 and G2 because of low starting signal in G1. This fluorescence was lost easily by photobleaching from eGFP and JF549 acquisition. Contrast has been enhanced in Scc1-Halo^{JF549} channel in post bleach (after soluble fraction was bleached intentionally) and G2 (as there was a decrease in total nuclear signal). Scale bar, 5 µm.
