# Supplementary figures and images for: Cohesin Can Remain Associated with Chromosomes during DNA Replication
Source: Cell Rep. 2017 Sep 19;20(12):2749–55. doi: 10.1016/j.celrep.2017.08.092 (PMC5613076; doi:10.1016/j.celrep.2017.08.092)

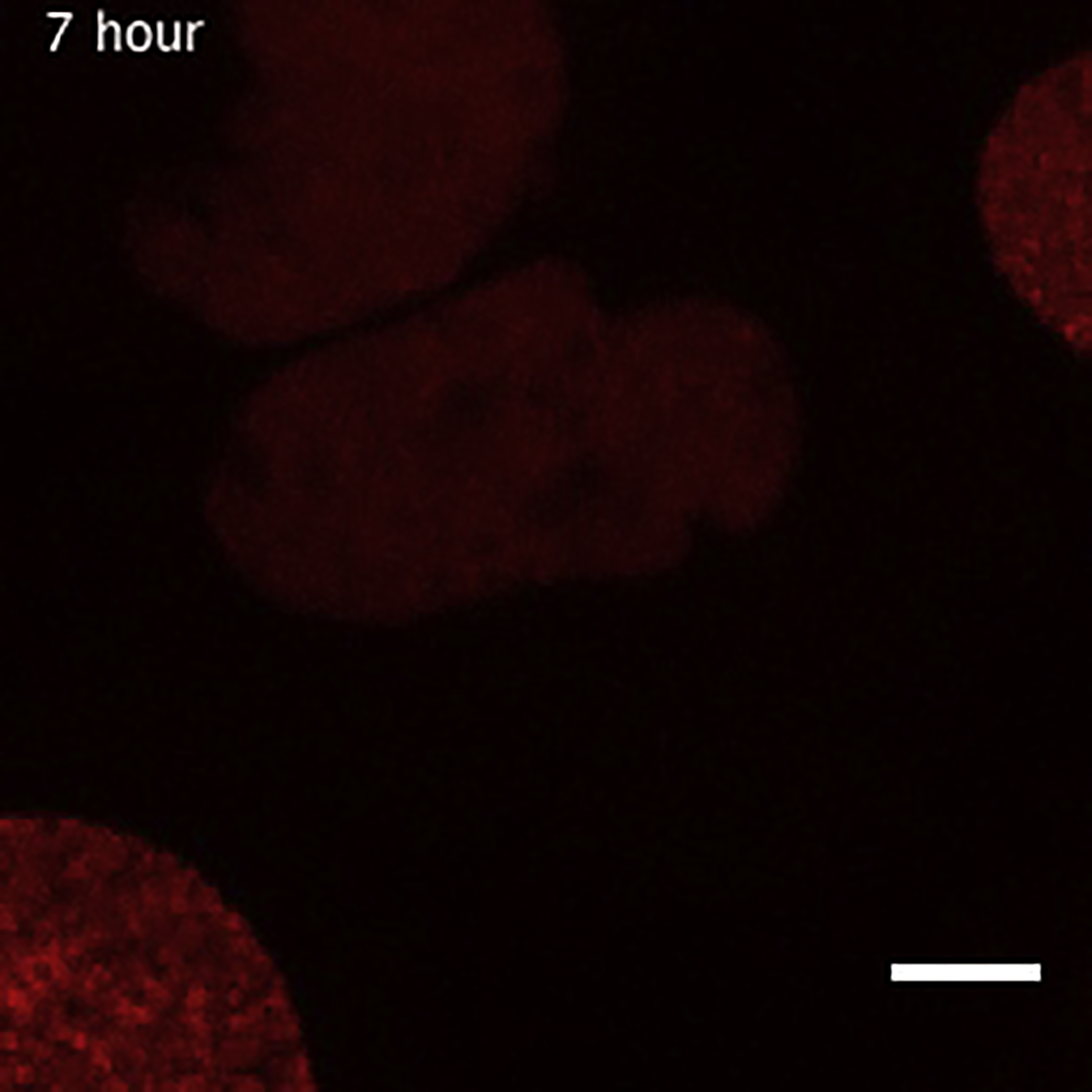

Supplement: Movie S1. Scc1 Is Rapidly Degraded after Anaphase in WAPL11161119Δ Cells — Live-cell microscopy movie of Scc1-HaloJF549WAPL1116-1119Δ cells undergoing mitosis in the presence of the non-fluorescent HaloTag ligand. [file mmc2.jpg]
